# Intranasal Delivery of a Methyllanthionine-Stabilized Galanin Receptor-2-Selective Agonist Reduces Acute Food Intake

**DOI:** 10.1007/s13311-021-01155-x

**Published:** 2021-12-02

**Authors:** Anneke Kuipers, Márta Balaskó, Erika Pétervári, Andreas Koller, Susanne M. Brunner, Gert N. Moll, Barbara Kofler

**Affiliations:** 1 Lanthio Health B.V., Rozenburglaan 13B, 9727 DL Groningen, Netherlands; 2 Institute for Translational Medicine, Medical School, University of Pécs, 12 Szigeti út, H-7624 Pécs, Hungary; 3 Research Program for Receptor Biochemistry and Tumor Metabolism, Department of Pediatrics, University Hospital of the Paracelsus Medical University, Muellner Hauptstr. 48, 5020 Salzburg, Austria; 4 Research Program for Experimental Ophthalmology, Department of Ophthalmology and Optometry, University Hospital of the Paracelsus Medical University, Salzburg, Austria; 5 Department of Molecular Genetics, Groningen Biomolecular Sciences and Biotechnology Institute, University of Groningen, Nijenborgh 7, 9747 AG Groningen, Netherlands

**Keywords:** Lanthionine, Ligand, GPCR, Neuropeptide, *Lactococcus*, Feeding

## Abstract

The regulatory (neuro)peptide galanin is widely distributed in the central and peripheral nervous systems, where it mediates its effects via three G protein-coupled receptors (GAL_1-3_R). Galanin has a vast diversity of biological functions, including modulation of feeding behavior. However, the clinical application of natural galanin is not practicable due to its rapid in vivo breakdown by peptidases and lack of receptor subtype specificity. Much effort has been put into the development of receptor-selective agonists and antagonists, and while receptor selectivity has been attained to some degree, most ligands show overlapping affinity. Therefore, we aimed to develop a novel ligand with specificity to a single galanin receptor subtype and increased stability. To achieve this, a lanthionine amino acid was enzymatically introduced into a galanin-related peptide. The residue’s subsequent cyclization created a conformational constraint which increased the peptide’s receptor specificity and proteolytic resistance. Further exchange of certain other amino acids resulted in a novel methyllanthionine-stabilized galanin receptor agonist, a G1pE-T3N-S6A-G12A-methyllanthionine[13–16]-galanin-(1–17) variant, termed M89b. M89b has exclusive specificity for GAL_2_R and a prolonged half-life in serum. Intranasal application of M89b to unfasted rats significantly reduced acute 24 h food intake inducing a drop in body weight. Combined administration of M89b and M871, a selective GAL_2_R antagonist, abolished the anorexigenic effect of M89b, indicating that the effect of M89b on food intake is indeed mediated by GAL_2_R. This is the first demonstration of in vivo activity of an intranasally administered lanthipeptide. Consequently, M89b is a promising candidate for clinical application as a galanin-related peptide-based therapeutic.

**Supplementary Information:**

The online version contains supplementary material available at 10.1007/s13311-021-01155-x.

## Introduction

Galanin is a 29 amino acid peptide that was first isolated from porcine intestine in 1983 [1] and was later found in many other species, displaying a high degree of conservation of the N-terminal part. Human galanin is unique in that it consists of 30 amino acids and, in contrast to other species, does not undergo amidation at the C-terminus.

Typical for regulatory peptides, galanin is derived from a 123 (human)/124 (murine) amino acid preproprecursor molecule. Proteolytic cleavage results in the mature galanin peptide. Consistent with the classic definition of neuropeptides, galanin is widely distributed in both the central and peripheral nervous systems; however, it is also found in non-neuronal peripheral tissues. In the 37 years since its discovery, a vast diversity of biological functions have been described for galanin, including roles in modulation of neurotransmission, nociception, learning and memory, anxiety and related behaviors, immunity and inflammation, neuronal injury and survival, regeneration, and neuroprotection (for review see [2, 3]). Recently, we demonstrated that galanin is a modulator of immune cell function [4–7]. Furthermore, central administration of galanin produced a rapid increase in food intake [8–12]. Acute effects of galanin on feeding were abolished by galanin receptor (GALR) antagonists [11]. Initially, neither GAL_1_R-KO nor GAL_2_R-KO mice were reported to display any marked phenotype related to differences in body weight or feeding behavior [13–15]. However, subsequent studies indicated that the endogenous galanin-GAL_1_R system plays a role in adjusting food intake and/or metabolism to acute changes in dietary fat [16, 17].

Galanin exerts its biological effects by signaling via G protein-coupled receptors (GPCRs). To date, three endogenous GALRs have been identified and termed GAL_1-3_R. Each receptor subtype shows high interspecies homology, but the three GALRs are only moderately homologous to each other. They differ in their tissue-specific expression pattern and, most importantly, in their functional coupling and signal transduction pathways, which contributes to the diversity of galanin-mediated effects [3]. GAL_1_R couples to G_i_-type G proteins, resulting in inhibition of cAMP production, opening of G protein-regulated inwardly rectifying K^+^ (GIRK) channels, and stimulation of mitogen-activated protein kinase (MAPK) activity. GAL_2_R signals through multiple classes of G proteins and stimulates multiple intracellular pathways; however, it preferentially couples to G_q/11_-type G proteins, leading to phospholipase C activation, which stimulates Ca^2+^ release via inositol phosphate hydrolysis and opens Ca^2+^-dependent ion channels in a pertussis toxin (PTX)-resistant manner. Evidence from various in vitro and in vivo studies also shows GAL_2_R coupling to G_i/o_- and G_12/13_-type G proteins. The signaling properties of GAL_3_R are still poorly defined. GAL_3_R appears to act mainly via PTX-sensitive G_i/o_-type G proteins, resulting in activation of GIRK channels as well as decreased adenylate cyclase activity and cytosolic cAMP levels. In addition, potential heteromerization of GALRs has been proposed [18, 19].

Since the discovery of the three GALRs, major effort has been directed at developing receptor-selective agonists and antagonists. These were based on substitution of certain amino acids in the galanin peptide sequence. While receptor selectivity was achieved to some degree, GAL(2–11) turned out to be a very selective non-GAL_1_R agonist. Most of the other analogs showed overlapping affinity [3]. We showed that Ala5-galanin(2–11) is effectively a GAL_2_R-specific agonist, with a more than 375-fold preference for GAL_2_R compared to both GAL_1_R and GAL_3_R [20].

The use of natural galanin in clinical applications faces two serious limitations: lack of receptor specificity and in vivo susceptibility to rapid peptidase-mediated breakdown. Endogenous galanin displays no receptor specificity, but its affinity to each receptor subtype varies (GAL_1_R > GAL_2_R > GAL_3_R) (for review see [2]). Interestingly, only the first 15 amino acids of the N-terminal part of the peptide are important for the interaction with galanin receptors. Recently, a selective GAL_2_R agonist based on the sequence of spexin, a newly discovered natural peptide with affinity for GAL_2_R and GAL_3_R, was reported to have a half-life of about 8 h in serum [21]. In vitro biostability studies revealed that the half-life of synthetic galanin in cerebrospinal fluid is 60 to 120 min [22]. The in vivo half-life of galanin in plasma is around 4 min [23, 24]. In view of this short in vivo half-life, galanin analogs with increased stability are needed to achieve therapeutic potential.

A promising alternative could be the introduction of lanthionines into galanin analogs, which may enhance receptor specificity and stability. Lanthionine (alanine-sulfur-alanine) is a highly stable, monosulfide bridge-containing amino acid. Cyclization by the methyllanthionine structure leads to a conformational constraint. Owing to this constraint, the receptor specificity may increase, and the peptide becomes resistant to breakdown by peptidases, leading to a prolonged in vivo half-life and potentially broader delivery options [25–28]. For example, a lanthionine-stabilized angiotensin-(1–7) has strongly increased resistance to peptidases and has shown therapeutic efficacy in animal models of cardiovascular, metabolic, kidney, and pulmonary disease [29–32].

For delivery of therapeutic peptides and proteins to the brain, the blood–brain barrier (BBB) is a major impediment. Intranasal delivery might provide a non-invasive means to bypass the BBB. Advantages of applying peptides via the nasal route, provided they do indeed reach the brain, include minimizing exposure to peripheral organs and tissues, thus reducing systemic side effects. It also allows peptides, which typically undergo both rapid degradation in the blood and excretion, sufficient time to exert their effect. Intranasal delivery of peptides may provide the ability to target specific regions of the brain when administered with substrates like cyclodextrins [33, 34]. Indeed, the use of cyclodextrins has been one of the most successful approaches to target peptides to various brain regions. Cyclodextrins have long been used in nasal drug delivery as adsorption-enhancing compounds to increase the intranasal bioavailability of protein and peptide drugs [33, 35]. Recent studies have revealed that intranasally applied galanin-like peptide (GALP), which is an endogenous ligand of GALRs, has a central anti-obesity action in addition to its role in food intake regulation [36]. Intranasal administration of GALP with α-cyclodextrins increased uptake in all brain regions by two- to threefold compared to GALP alone [36].

With the overall goal of developing galanin-based peptidergic drugs for clinical applications, the aim of the present study was to create a stable galanin analog with strict specificity to a single GALR subtype. We hypothesized that introduction of a pyroglutamate and methyllanthionine into galanin would enhance the peptide’s receptor specificity and stability.

Here, we report on the introduction of a methyllanthionine into C-terminally truncated galanin analogs by employing *Lactococcus lactis* bacteria that contain methyllanthionine-installing enzymes. To protect the peptides against breakdown by most aminopeptidases, a pyroglutamate was induced [37]. From 49 produced peptides, the G1pE-T3N-S6A-G12A-methyllanthionine[13–16]-galanin-(1–17) variant, termed M89b, was selected as our lead compound. M89b displays enhanced in vitro stability and unique GAL_2_R specificity. As a neuropeptide analog, we evaluated the in vivo activity of M89b following intranasal delivery.

## Methods

### Galanin Peptides

GAL-(1–15) was purchased from Tocris (Bio-Techne) and dissolved in water. For the production of methyllanthionine-stabilized galanin variants, *Lactococcus lactis* (*L. lactis*) harboring the two plasmids pIL3BTC and pNZM89bNdei was cultured. Plasmid pNZM89bNdei encodes the sequence for the substrate peptide fusion used for M89b. For example, for the case of M89b, this fusion peptide is composed of three parts, (1) the N-terminal 23 amino acid nisin leader peptide, (2) the export-enhancing N-terminal nisin-related part ITSISLCTPGCKTGAHMIE ending with a glutamate to allow cleavage by Glu-C, and (3) the C-terminally truncated galanin mutant **QWNLNAAGYLLATHACG**, which contains G1Q, T3N, S6AG12A, P13T, and V16C substitutions: MSTKDFNLDLVSVSKKDSGASPR-ITSISLCTPGCKTGAHMIE-QWNLNAAGYLLATHACG. Plasmid pIL3BTC encodes the genes for the lanthionine-installing enzymes NisB, which harbors the genes for the lanthionine-installing enzymes dehydrates threonine13 yielding dehydrobutyrine13, and NisC, which subsequently catalyzes the coupling of dehydrobutyrine13 to cysteine16 to form a methyllanthionine, and the transporter NisT, which exports the modified peptide into the culture medium [37].

*L. lactis* pIL3BTC pNZM89bNdei was grown overnight at 30 °C in M17 broth [38] supplemented with 0.5% glucose and chloramphenicol (5 µg/mL) and erythromycin (5 µg/mL). The overnight culture was diluted 100-fold in minimal medium [39] supplemented with glucose. To induce transcription of the genes encoding the fusion peptide, modification enzymes and the transporter, the nisin promoter-controlled nisin leader peptide galanin fusion and modifications and to export enzymes, the supernatant of the nisin-producing strain NZ9700 (1:1000) was added, and the culture was grown further for 24 h at 30 °C. Cell-free supernatant from the 4-L culture was equilibrated with 1 volume 100 mM lactic acid. Peptides (4 × 5 ml) were bound to HiTrap SP columns (GE Healthcare), washed with 50 mM lactate buffer pH 4, and eluted with 50 mM lactate buffer containing 1 M NaCl and 6 M urea. The eluted fractions were desalted by passage over PD-10 gel filtration columns (GE Healthcare). To cleave off the formed lanthi-galanin, the isolated fusion peptides were incubated in 100 mM phosphate buffer pH 7.6 supplemented with 250 U endoproteinase Glu-C V8 Protease (Merck) overnight at 37 °C. During the latter incubation, the N-terminal glutamine of the liberated peptide was largely converted into pyroglutamate. The released M89b peptide (and similarly other released peptides) was purified by RP HPLC with a C12 Proteo, 10 µm, 250 × 21.20 mm column (Jupiter) using an HP 1050 HPLC system (Agilent). M89b (and similarly other peptides) was collected, after which an additional HPLC run was performed for further purification. The purified M89b peptide (and similarly other peptides) was quantified by HPLC analysis with an RP C12 Proteo, 4 µm, 250 × 4.6 mm column (Jupiter) using a Jasco HPLC system, by comparing the A_280_ area of the peak with the A_280_ area of a known amount of galanin-(1–15) (Tocris). All HPLC runs were performed using a gradient of 10–90% acetonitrile (ACN) in 0.1% trifluoroacetic acid (TFA).

For the lanthipeptides used in the assays, a 500 µM stock solution in 25% ACN/0.05% acetic acid was prepared from which dilutions were made in water.

### cAMP Assay

To evaluate the ability of lanthipeptides to induce cAMP production via GAL_1_R signaling, we used the cAMP Hunter™ eXpress GAL_1_R CHO-K1 GPCR Assay from Eurofins-DiscoverX. The assay was performed according to the manufacturer’s instructions. Briefly, for one assay, CHO-K1 cells expressing GAL_1_R were diluted in AssayComplete™ Cell Plating 2 Reagent, and 100 µl was seeded into each well of a 96-well plate. The plate was incubated at 37 °C, 5% CO_2_ in a humidified incubator. After 24 h of incubation, the media was aspirated, and 45 µl cell assay buffer/antibody mixture was added to all wells. For the inhibition of intracellular cAMP accumulation, induced by forskolin, a serial threefold dilution of the lanthipeptides (10 µM to 5 nM) in 80 µM forskolin was made. Fifteen µl of the serial dilutions was applied in duplicates to the cells. The concentration of forskolin in the assay was 20 µM. The agonists were incubated for 30 min at 37 °C. Next, 15 µL Antibody Solution and 60 μL cAMP Working Detection Solution were added, and the plate was incubated for 1 h in the dark at room temperature. Finally, 60 μL cAMP Solution A was added and, after 3 h of incubation in the dark at room temperature, the chemiluminescent signal was read with a Synergy HT Biotek reader.

### ß-Arrestin Assay

To evaluate the ability of lanthipeptides to recruit ß-arrestins via GAL_1_R or via GAL_2_R, we used the PathHunter® eXpress GAL_1_R, CHO-K1, β-arrestin GPCR Assay and the PathHunter® eXpress GAL_2_R, CHO-K1, β-arrestin, GPCR Assay from Eurofins-DiscoverX. The assays were performed according to the manufacturer’s instructions. Briefly, for one assay, CHO-K1 cells co-expressing GAL_1_R or GAL_2_R and β-arrestin (isoform 2) were diluted in AssayComplete™ Cell Plating Reagent, and 100 µl was seeded into each well of a 96-well plate. The plate was incubated at 37 °C, 5% CO_2_ in a humidified incubator. A serial 3 × dilution of the lanthipeptides in water was made (10 µM to 5 nM). After 48 h of incubation, 10 µl of the serial dilutions was applied to the cells in duplicate. The agonists were incubated for 90 min at 37 °C. Next, 55 µL of Working Detection Solution was added, and after 1 h of incubation at room temperature in the dark, the chemiluminescent signal was read with a Synergy HT Biotek reader.

### Fluo-4 Direct Calcium Assay

Calcium efflux was measured by using a Fluo-4 Direct Calcium Assay purchased from Thermo Fisher Scientific. HEK293 cells with stable overexpression of GAL_2_R (HEKR2) (kindly provided by Harald Dargatz, Molecular, Cellular and Pharmacobiology Section, Institute of Pharmaceutical Biology, University of Bonn, 53,115 Bonn, Germany) were used for analysis of the lanthipeptides. HEKR2 cells were seeded in a 96-well plate and grown overnight in DMEM supplemented with 10% FBS, 1% penicillin–streptomycin, 100 µg/ml Normocin, and 500 µg/ml G418 at 37 °C, 5% CO_2_ in a humidified incubator.

The next day, when the cells were nearly confluent, an equal amount of 2 × Fluo-4 Direct™ calcium reagent loading solution, containing 5 mM probenecid, was added to the wells, and the plate was incubated for 1 h at 37 °C. A serial 3 × dilution of the agonist compound in water was made, and 10 µl of the serial dilutions was applied to the cells in duplicate. The starting concentration of agonist in the assay was 10 µM. After 1–2 h of incubation at room temperature, the fluorescence was measured with excitation at 485/20 nm and emission at 528/20 nm with a Synergy HT Biotek reader.

### Label-Free Dynamic Mass Redistribution Assay

SH-SY5Y cells stably expressing low levels of either human GAL_1_R (SY5Y-R1) or GAL_2_R (SY5Y-R2) and HEK293 cells expressing human GAL_3_R (HEK-R3) [39, 40] were seeded in EnSpire LFC-348 well plates (Perkin Elmer) at a concentration of 12,000 cells per well and cultured overnight at 37 °C and 5% CO_2_. The medium was aspired, and the cells were washed four times with Hank’s balanced salt solution (HBSS) (+ calcium, + magnesium) supplemented with HEPES (20 mM), and the final volume was adjusted to 30 µl per well. The plate was allowed to equilibrate to assay temperature for 1 h in the EnSpire multimode reader (Perkin Elmer). Galanin-(1–30) (GL Biochem) was dissolved and diluted in water. Five-hundred µM stock solutions of M50b, M54, M74b, and M89b were prepared in 25% ACN/0.05% acetic acid from which dilutions were made in water. HBSS (+ / +) buffer supplemented with HEPES (20 mM) alone or a dilution series of galanin, M50b, M54, M74b, and M89b (1.6 nM to 5 µM) dissolved in the same buffer was prepared and placed on a compound plate which was allowed to equilibrate to reach the same temperature as the EnSpire plate holding the cells. A baseline read was first generated by taking 4 measurements before ligands were added. The plate was measured between 30 and 70 repeats, and each treatment was performed in triplicate. The experiment was repeated two times for M50b and M74 and three times for M89b, M54, and galanin. EC_50_ values were calculated using Prism 8.2.0 (GraphPad Software Inc.). The values are provided as means of all independent experiments.

### Luciferase Reporter Assay

The luciferase reporter assay uses a reporter plasmid and a receptor plasmid transiently transfected into the target cell line. The reporter plasmid contains a serum response element (SRE) coupled to a luciferase open reading frame and induces expression of the luciferase upon G protein activation. Genetically modified HEK293 G_qi_ cells [41] were transiently transfected with the SRE reporter and the receptor (GAL_1-3_R) plasmids as described previously [21, 42]. HEK293 G_qi_ cells and plasmids were kindly provided by Young Seong (Graduate School of Medicine, Korea University, Seoul, Republic of Korea). The cells were treated with full-length galanin (prepared as a 1 mM stock in water containing 0.1% BSA and diluted with the same vehicle) and M89b (500 µM stock solutions were prepared in 25% ACN/0.05% acetic acid/0.1% BSA from which dilutions were made in water containing 0.1% BSA) (0.1 nM to 10 µM) for 5 h. The luciferase activity was analyzed with the Bright-Glo Luciferase Assay (Promega) in the EnSpire plate reader. The experiment was repeated three times. EC_50_ values were calculated using Prism 8.2.0 (GraphPad Software Inc.) and are provided as means of all independent experiments.

### In Vitro Stability in Rat Serum

The stability of M89b was assessed by incubation of 10 µM of the N-terminal fragment 1–15 of linear galanin (GAL-(1–15)) and 10 µM M89b in 5% rat serum buffered with 20 mM phosphate buffer (pH 7.4) at 37 °C for up to 23.5 h. At various time points (0, 30, 90, 270, and 1410 min), 180 µl of samples were quenched with 10 µl 10% TFA and 20 µl 100% ACN. Samples were centrifuged and kept on ice. The amount of full-length peptides in the serum was analyzed using a C12 column (C12 RP 250 × 4.60 mm column, Phenomenex) on a JASCO HPLC system and applying a gradient of 10–90% ACN in 0.1% TFA for 45 min. Peptides were detected at 280 nm. A_280_ areas in chromatograms of the corresponding GAL-(1–15) and M89b full-length peptide peaks were used to determine the amount of peptide left after incubation with 5% rat serum at the different time points. Peptide peaks were determined using JASCO-Borwin Chromatography software (v1.50).

### In Vitro Stability in Cerebrospinal Fluid

Pooled male Wistar Hannover rat cerebrospinal fluid was purchased from BioIVT (West Sussex, UK). Stability of peptides was analyzed by incubation of 4 µl 0.50 mM GAL-(1–15) with 10 µl cerebrospinal fluid or 6 µl 0.33 mM M89b with 10 µl cerebrospinal fluid at 37 °C [43]. At various time points (0, 30, 60, 240, and 480 min), the reactions were quenched with 2 µl 50% TFA and 20 µl 100% ACN. Water was added to reach a final volume of 220 µl. Samples were centrifuged and kept on ice. Full-length peptides were quantified by using a C12 column (C12 RP 250 × 4.60 mm column, Phenomenex) on a JASCO HPLC system and applying a gradient of 20–90% ACN in 0.1% TFA for 30 min. Peptides were detected at 280 nm. A_280_ areas in chromatograms of the corresponding GAL-(1–15) and M89b full-length peptide peaks were used to determine the amount of peptide left after incubation with cerebrospinal fluid at the different time points. Peptide peaks were determined using JASCO-Borwin Chromatography software (v1.50).

### In Vitro Stability in Brain Plasma

The method to study the stability of peptides in brain plasma is described in the Supplementary material.

### In Vivo Pharmacokinetics

Pharmacokinetics studies were performed at Pharmidex UK. The animal experimental protocols were approved by the UK Government Home Office and carried out in accordance with the guidelines of the Animals (Scientific Procedures) Act (1986). Ethical approval was subject to UK Home Office license number P651A96A4.

One mg HPLC-purified and HPLC-quantified M89b was dissolved in 1 ml Milli-Q followed by thorough vortexing and warming the solution for 10 min to 60 °C. From this solution, a dilution in 0.1% 2-hydroxypropyl-β-cyclodextrin was made. Male CD1 mice (weighing 26.5 to 32.9 g, *n* = 3 per time point) were used for the pharmacokinetic studies. Mice were given 1 mg/kg M89b either subcutaneously (s.c.) or intravenously (i.v.). Samples were collected after 2, 5, 10, 15, 30, 60, 120, and 240 min. Post-dosing, the 4 h mice were individually housed in metabolic cages for cold collection of urine in pre-weighed tubes which were re-weighed prior to storage. At each scheduled sampling time point, terminal blood was collected from each animal by cardiac puncture in heparin-containing tubes (20 IU heparin/mL of blood) kept on wet ice. The blood was centrifuged (5 min at 21, 100 g at 4 °C), and the resulting plasma was transferred to the corresponding labeled polypropylene tubes prior to storage. Following blood collection, the mice were euthanized, and the brain was transferred to pre-weighed labeled polypropylene tubes which were re-weighed prior to storage. All samples were snap frozen and stored at − 70 °C prior to analysis.

### Preparation of Peptide Solutions for Intranasal Delivery

For intranasal delivery, 1 mg of M89b was first dissolved in 566 µl 2,2,2-trifluorethanol, 566 µl 10% (2-hydroxypropyl)-ß-cyclodextrin (HBC), and 1 µl acetic acid and incubated at 60 °C for 20 min. Another 566 µl of 10% HBC were added. The peptide was then dried in a speed-vac, and the dried pellet was dissolved in 1132 µl water by vortexing and incubation at 60 °C. M871 and GALP were purchased from GL Biochem. M871 was dissolved in saline containing 10% HBC. GALP was dissolved in saline containing 5% α-cyclodextrin.

### Food Intake Measurement After Intranasal Application of Peptides

In vivo experiments involving intranasal treatment of rats and measurement of feeding behavior were performed at the Institute for Translational Medicine, University of Pécs, Pécs, Hungary. Experiments were approved by the National Ethical Council for Animal Research (permit number, BA 02/2000–11/2018). They were also in accord with the directives of the European Communities Council on the protection of animals used for scientific purposes (86/609/EEC, Directive 2010/63/EU of the European Parliament and of the Council).

Spontaneous food intake was measured in non-fasted male Wistar rats (3 months old) for 24 h following intranasal application of peptide solutions by an automated FeedScale System (Columbus, OH). Each group of animals (6–9 rats/group) was habituated to the individual chambers of the FeedScale System until daily food intake and changes in the body weight of the animals had normalized. This habituation, which lasted for a minimum of 2 weeks, allowed adaptation to the environment, to the powdered chow and to the feeder. This powdered form of chow prevented food hoarding. A special digital scale under the cages provided precise automated measurement and continuous recording of the amount of consumed food. During the measurement of the cumulative 24 h food intake, data were collected every 30 min.

Intranasal applications were performed at 06:00 PM at the onset of the active nighttime period of rats. First, the body weight of animals was recorded, and then they were intraperitoneally anesthetized with ketamine (52 mg/kg) and xylazine (9 mg/kg). For the intranasal application of peptides, rats were placed on their backs, and 25–30 µl of peptide solution was injected gently per nostril (total volume of 50–60 μl per rat depending on the administered peptides) using a 10-μl pipette. The following solutions were applied: M89b (25 nmol in 60 µl saline containing 10% HBC); GALP (17.5 nmol in 50 µl saline containing 5% α-cyclodextrin); M871 (219 nmol in 60 µl 10% HBC); and M89b + M871 (25 nmol M89b + 219 nmol M871 in 60 µl 10% HBC). Control rats received the same volume of vehicle only. The total 50–60 μl of solution per rat was given in small drops of approximately 5 μl/drop over a 20 min period, alternating drops every 2 min between the left and right nares. Complete recovery from the effects of anesthesia usually required 2 h. Following intranasal treatment, animals were placed into their individual chamber of the FeedScale System, and measurement of the food intake was started. At 24 h post-treatment, the body weight of the animals was measured.

### Statistical Analysis

Statistical analysis was performed with SPSS 11.0. Graphs were created using GraphPad Prism 9.0. Repeated-measures two-way ANOVA with Tukey’s post hoc test was applied for the statistical analysis of the food intake data. Body weight data were analyzed by applying a one-way ANOVA with Tukey’s post hoc test. Differences were accepted as statistically significant at the level of *p* < 0.05. Mean ± S.E.M. are indicated in all figures.

### Data availability

The raw data supporting the conclusions of this article will be made available by the authors, without undue reservation, to any qualified researcher.

## Results

### Design of Methyllanthionine-Stabilized Galanin Variants

To develop a stable galanin-related variant with single GALR specificity, we introduced methyllanthionines into a C-terminally truncated galanin-related mutant, making use of bacteria containing methyllanthionine-introducing enzymes. These enzymes dehydrate threonine residues and stereospecifically couple the formed dehydrobutyrine to cysteine, after which the formed D,L-(methyl)lanthi-galanin variants, also called lanthi-galanins belonging to the class of lanthipeptides, are exported into the bacterial culture medium [44–46]. By mutagenesis, the positions of the threonine and cysteine were varied to allow the production of a large number of enzyme-installed methyllanthionine-containing galanin variants.

As an initial screen, the biological activity of all produced variants was determined by using CHO-K1 cell lines expressing either GAL_1_R or GAL_2_R in commercial β-arrestin recruitment (GAL_1_R or GAL_2_R) and cAMP assays (GAL_1_R). In addition, human embryonic kidney 293 (HEK293) cell lines that express GAL_2_R were used to measure Ca^2+^ efflux. GAL_1_R and GAL_2_R were chosen because of their therapeutic potential in a range of different diseases and their involvement in appetite regulation [3].

Enzymatic introduction of a methyllanthionine consists of two steps: (1) dehydration of an engineered threonine yielding dehydrobutyrine and (2) coupling of the formed dehydrobutyrine to an engineered cysteine, thus yielding methyllanthionine. To obtain one product, instead of a mixture of peptides resulting from partial dehydration, the mutations T3N and S6A were introduced, and the peptide was found to retain significant GALR activity (Table 1). Therefore, these T3N and S6A mutations were retained in all subsequent variants.

**Table 1:**
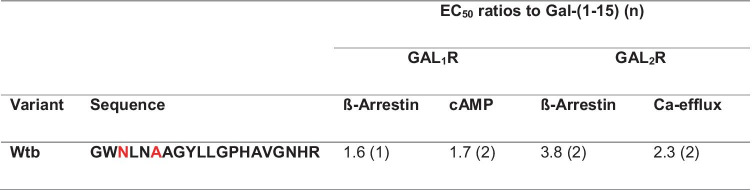
Ratios of the half-maximal effective concentration (EC_50_) of the T3N-S6A galanin variant in comparison to Gal-(1–15) in activity assays using GAL_1_R- and GAL_2_R-transfected cell lines

Methyllanthionine introduction N-terminal to position 13 abolished GALR activity (Table 2). This is consistent with the fact that these N-terminal amino acids of galanin are essential for GALR activity [2]. Consequently, methyllanthionine introduction from position 13 onwards was better tolerated and allowed for varying GALR specificity (Table 3).

**Table 2:**
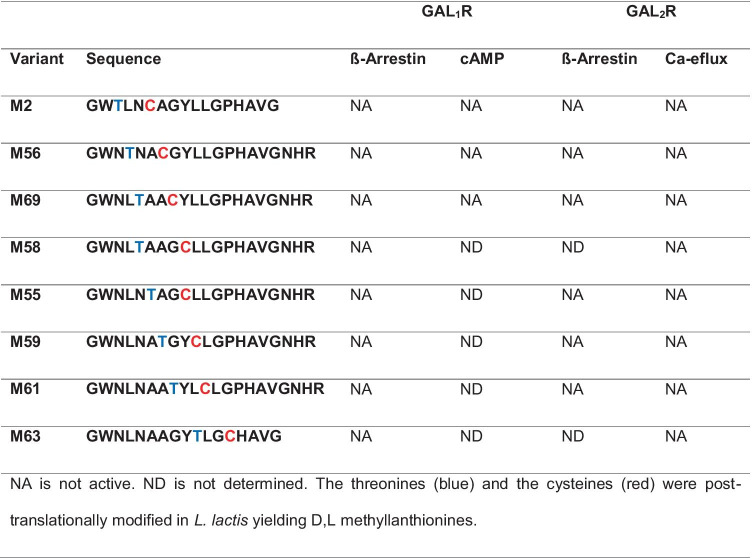
Ratios of the half-maximal effective concentration (EC_50_) of the methyllanthionine-containing galanin variants in comparison to Gal-(1–15) in activity assays using GAL_1_R- and GAL_2_R-transfected cell lines

**Table 3:**
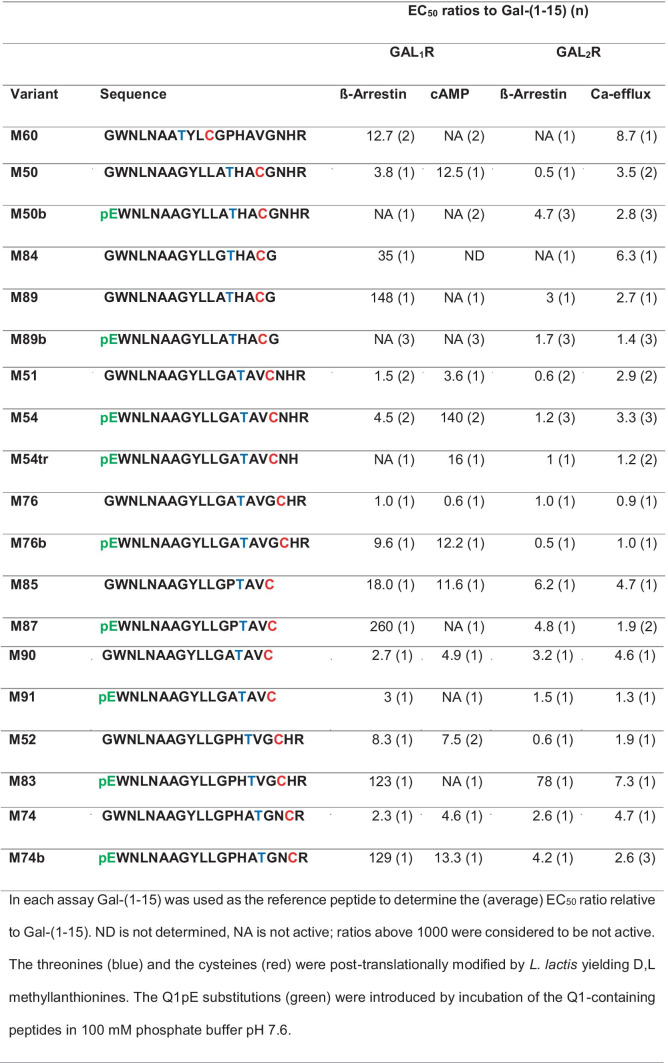
Ratios of the half-maximal effective concentration (EC_50_) of the methyllanthionine-containing galanin variants in comparison to Gal-(1–15) in activity assays using GAL_1_R- and GAL_2_R-transfected cell lines

Therefore, to keep the active lanthipeptide short, we aimed to produce methyllanthionine [13–16]. For the formation of a methyllanthionine [13–16], engineering of a dehydratable threonine at position 13 and a cysteine at position 16 is required. Since an alanine directly flanking threonine favors its dehydration by the nisin dehydratase, a G12A mutation was also introduced. These mutations allowed the enzymatic formation of dehydrobutyrine13 and its enzymatic coupling to cysteine16 to yield a D,L-[13–16]methyllanthionine [47, 48].

Pyroglutamate (pE) is a natural amino acid derivative in which the free amino group of glutamine cyclizes to form a lactam. Introduction of pE at position 1 protects against degradation by aminopeptidases [37] other than pyroglutamate aminopeptidase [49]. Therefore, a G1Q mutation was introduced for subsequent pE formation. In addition, N-terminal pE seems to strongly contribute to the preference of some analogs (e.g., M50b, M74b, and M89b) but not all (e.g., M76b), for GAL_2_R (Table 3).

**Fig. 1:**
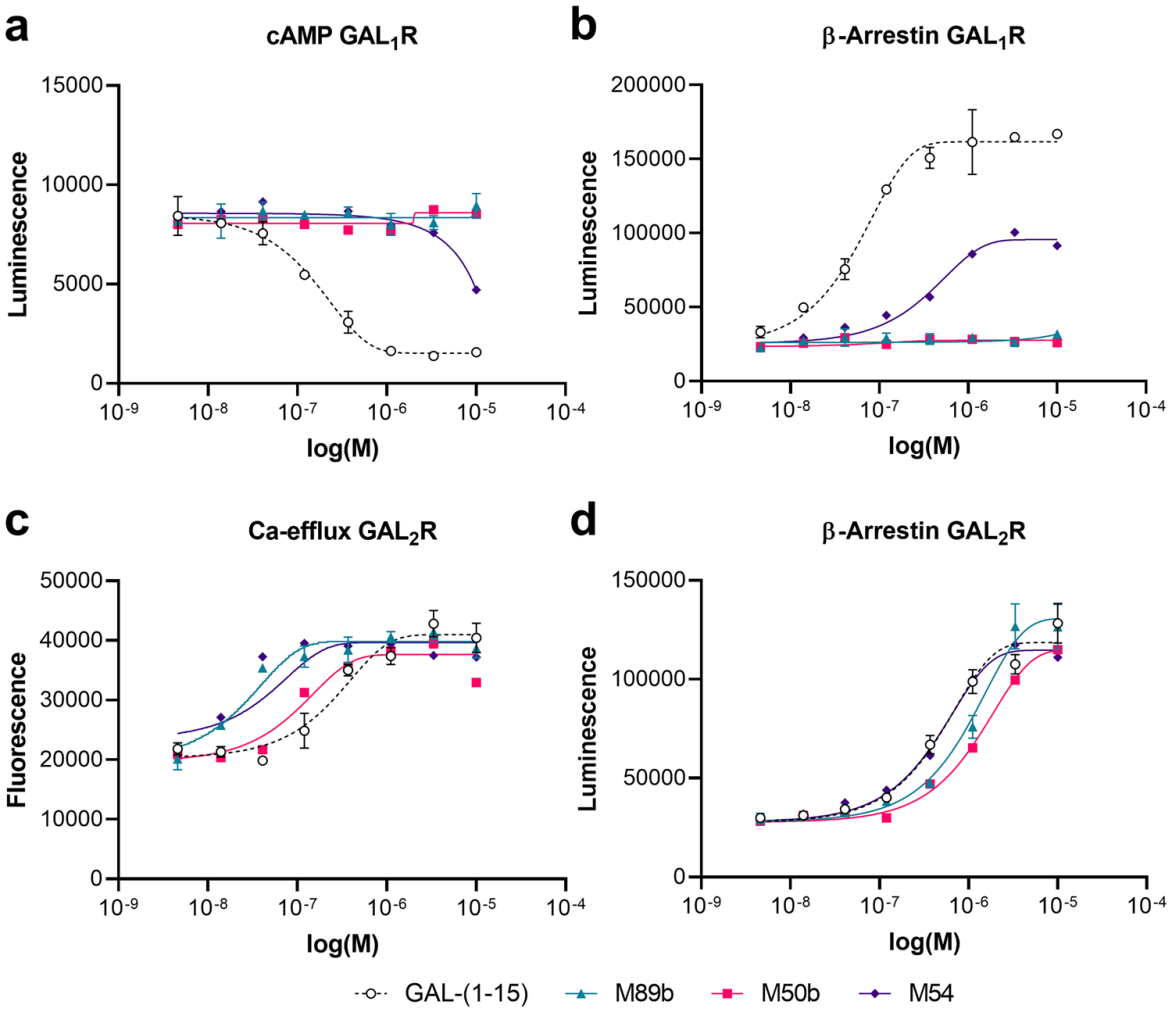
M89b only stimulates GAL_2_R but not GAL_1_R signaling. GAL_1_R-expressing CHO-K1 cells (**a**, **b**) and GAL_2_R-expressing HEK293 cells (**c**) or CHO-K1 cells (**d**) were used to test the ability of GAL-(1–15), M89b, M50b, and M54 (5 nM to 10 µM) to modulate cAMP-related luminescence (LU) (**a**), ß-arrestin-related (**b**, **d**), or Ca^2+^-efflux related fluorescence (**c**). Data represent mean ± SEM (*n* = 2)

Among the peptide variants we produced, especially M50b, M54, M74b, M87, and M89b showed a preference for stimulating GAL_2_R. While the variants M54, M74b, and M87 retained some activity toward GAL_1_R, both M50b and M89b showed complete absence of activity toward GAL_1_R-expressing cells. Importantly, M89b, a shorter peptide than M50b and thus less costly for eventual chemical synthesis, showed slightly higher activity in the β-arrestin recruitment and cAMP assays compared to M50b (Fig. 1, Table 3).

### Receptor Subtype Selectivity of M89b

**Fig. 2:**
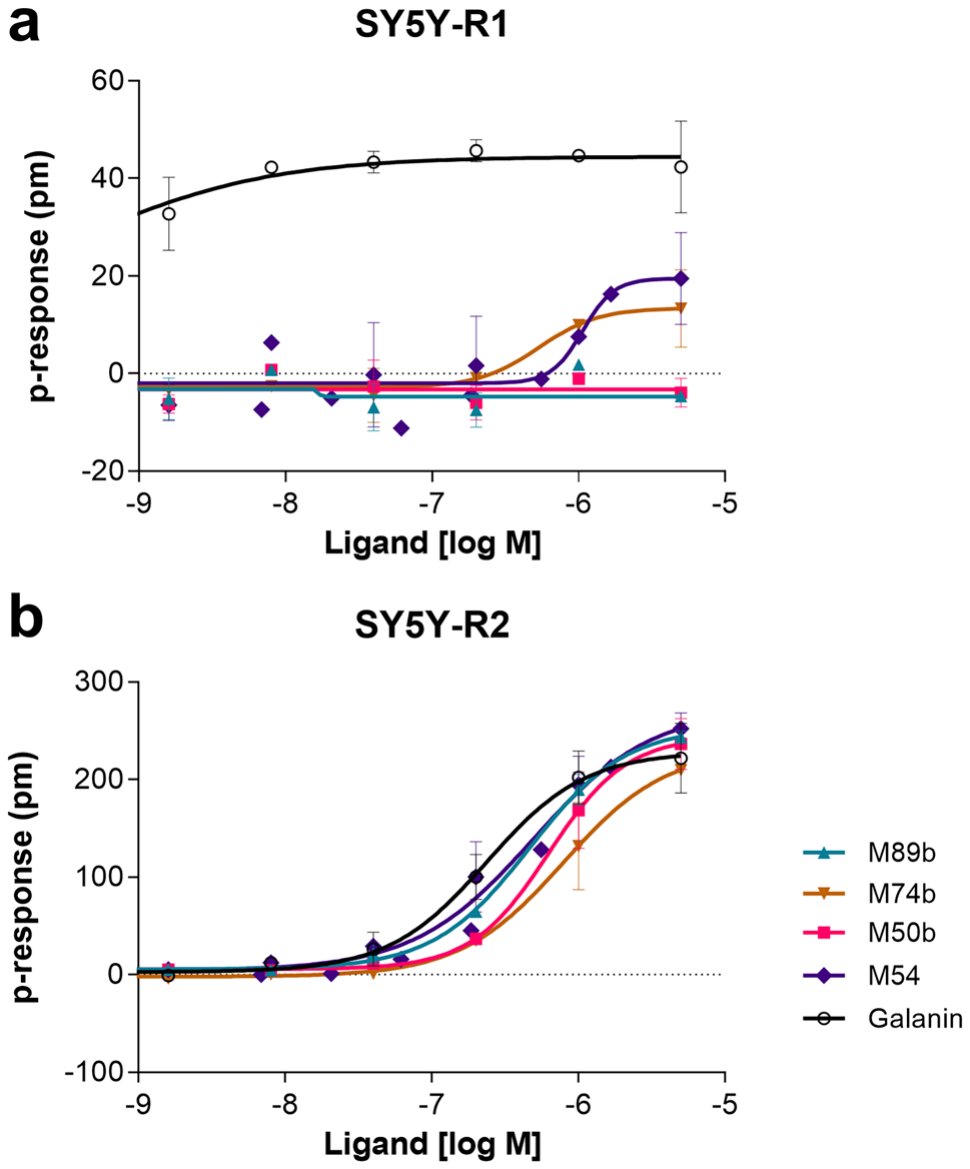
Dynamic mass distribution (DMR) of GAL_1_R- (**a**) and GAL_2_R- (**b**) expressing SY5Y cells upon treatment with lanthionine-stabilized galanin analogs and full-length galanin. Representative graphs showing dose-dependent change of DMR upon treatment with full-length galanin, M50b, M54, M74b, and M89b. Values represent means ± SEM (*n* = 1–3)

**Fig. 3:**
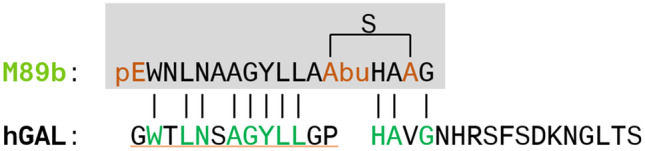
The sequence of M89b. The sequence of the methyllanthionine-stabilized galanin analog, M89b, in comparison to full-length human galanin (green letters indicate sequence homologous amino acids between M89b and galanin; pE, pyroglutamate; Abu-S-Ala is a methyllanthionine; Abu is aminobutyric acid)

In the label-free assay, full-length galanin induced a change of the dynamic mass distribution (DMR) in GAL_1_R- and GAL_2_R-transfected cell lines (SY5YR1 and SY5YR2), confirming lack of selectivity of galanin toward GALR subtypes (Fig. 2a, b). By measuring the ability of lanthipeptides to modulate the DMR of SY5YR1 and SY5YR2 cells, we confirmed that M50b and M89b stimulate GAL_2_R only, not GAL_1_R; this is in contrast to M54 and M74b, which induced a very limited DMR also in SY5YR1 cells (EC_50_ 1.1 µM and 535 nM, respectively) (Fig. 2a, b). Furthermore, the half-maximal effective concentration of M89b toward GAL_2_R was higher (EC_50_ 562 nM) compared to galanin (EC_50_ 255 nM) but was slightly lower compared to that of M50b (629 nM) (Fig. 2b). Therefore, we evaluated the activity, selectivity, and functionality of M89b, the G1pE-T3N-S6A-G12A-methyllanthionine[13–16]-galanin-(1–17) variant, in more detail (Fig. 3).

**Fig. 4:**
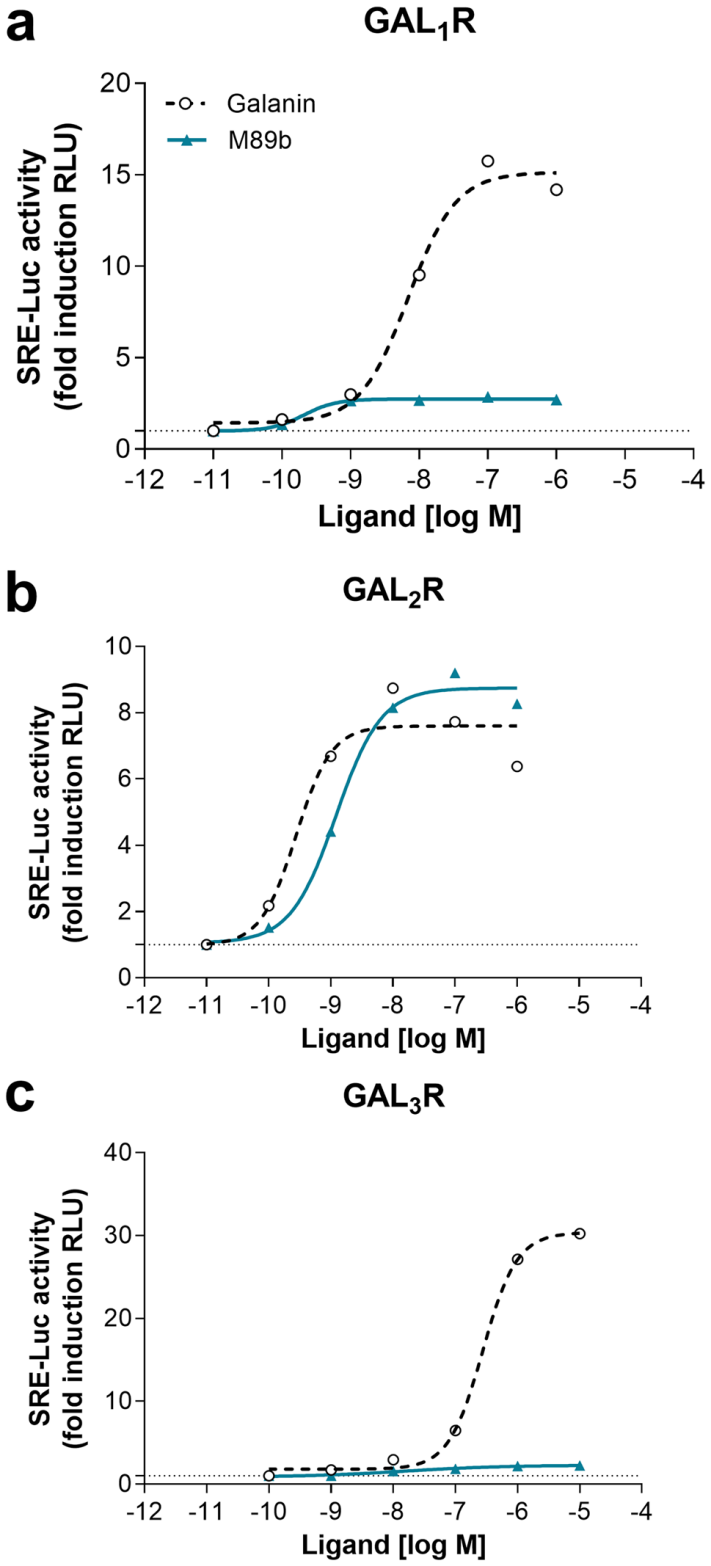
Luciferase reporter assay with GAL_1-3_R transiently transfected HEK293 G_qi_ cells. Graphs show representative luciferase reporter assays (*n* = 3) on GAL_1-3_R-transfected HEK293 G_qi_ cells upon treatment with full-length galanin and M89b. Luciferase activity was measured after 5 h of treatment with galanin or M89b in GAL_1_R- (**a**), GAL_2_R- (**b**), and GAL_3_R-transfected cells (**c**)

Using a label-free biochip technology, we were not able to observe activation of GAL_3_R by galanin in HEK293 cells following doxycycline-induced overexpression of GAL_3_R (HEKR3) [40]. One possible explanation is that, although we observed GAL_3_R mRNA expression in stably transfected HEKR3 cells (data not shown), the GAL_3_R was not expressed on the cell surface but remained within intracellular inclusion bodies [50]. This is supported by our observation of intracellular immunoreactivity of a GAL_3_R antibody in HEKR3-expressing cells [51]. Therefore, we used a luciferase assay as another reporter system and transiently GALR-expressing cells, in which GALRs are known to be expressed on the cell surface, to detect galanin signaling via GAL_1-3_R [42]. Galanin induced luciferase activity in all three (GAL_1-3_R) transiently transfected cell lines, with mean EC_50_ values of 9.7 nM (GAL_1_R), 1.0 nM (GAL_2_R), and 828 nM (GAL_3_R) (Fig. 4a–c). The luciferase reporter assay confirmed the specificity of M89b for GAL_2_R (EC_50_ 3.7 nM) (Fig. 4b) and its inability to stimulate GAL_1_R and GAL_3_R (Fig. 4a, c).

### M89b Has Increased In Vitro and In Vivo Stability

**Fig. 5:**
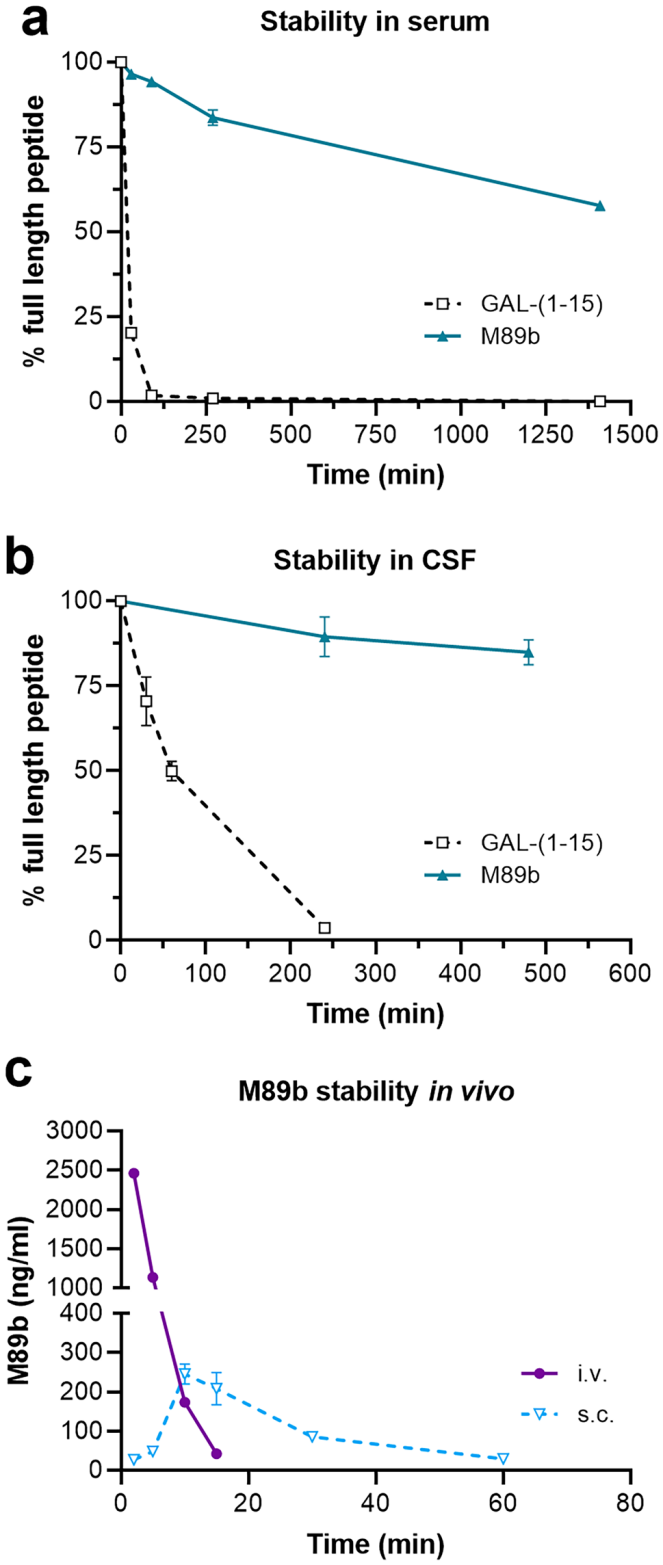
M89b has increased in vitro and in vivo stability. Degradation of M89b in comparison to GAL-(1–15) in 5% rat serum (**a**) and cerebrospinal fluid (**b**) over time and in vivo plasma levels of M89b after intravenous (i.v.) and subcutaneous (s.c.) injection of 1 mg/kg M89b to mice (**c**). Data represent means ± SEM. *n* = 2–3

In vitro stability of M89b and the galanin fragment GAL-(1–15) was tested in 5% rat serum, cerebrospinal fluid (CSF), and brain plasma. Remarkably, in rat serum after more than 23 h, only 40% of M89b had been degraded, and almost 60% of full-length M89b was retrieved. In contrast, already after 30 min, 80% of GAL-(1–15) was degraded, and after 90 min, less than 2% of GAL-(1–15) was detectable (Fig. 5a). In CSF, less than 4% of GAL-(1–15) was retrieved after 4 h, whereas almost 80% of full-length M89b were detected after 8 h (Fig. 5b). Finally, in brain plasma, already after 1 h, the whole amount of GAL-(1–15) was degraded, while almost 85% of full-length M89b were still detected after 6 h incubation (Supplementary Figure S1). Taken together, these data demonstrate the significantly prolonged in vitro half-life of M89b in various matrices.

Next, we analyzed the pharmacokinetics of M89b. M89b (1 mg/kg) was applied intravenously (i.v.) or subcutaneously (s.c.) to mice, and plasma levels of M89b were measured up to 4 h after administration. The in vivo half-life of M89b showed marked differences after i.v. and s.c. administration (Fig. 5c). While already 30 min after i.v. application no M89b was detectable in mouse plasma, low M89b plasma levels were measured at 60 min following s.c. administration. Importantly, M89b was not detected in urine samples at 4 h post i.v. or s.c. injection nor in brain tissue after s.c. administration and sampled at the same time points (data not shown). This suggests that peripherally administered M89b does not cross the BBB and is not immediately secreted via the kidneys.

### Intranasally Delivered M89b Inhibits Acute Food Intake and Reduced Body Weight via GAL_2_R

The GAL_2_R specificity of M89b suggests it could have therapeutic potential especially when delivered to the brain. To circumvent the BBB, we examined whether M89b could be delivered intranasally, a non-invasive route of administration [52]. As galanin peptides have been shown to modulate feeding, we tested the effect of M89b on acute food intake in rats. Since the efficacy of intranasal application of galanin-like-peptide (GALP) was enhanced when the peptide was complexed with α-cyclodextrin [36], we decided to use cyclodextrins as well to enhance intranasal delivery and stability of the peptides. GALP was applied in a 5% α-cyclodextrin solution. M89b is very hydrophobic, but we found improved solubility could be reached in 10% (2-hydroxypropyl)-ß-cyclodextrin (HBC) [53].

**Fig. 6:**
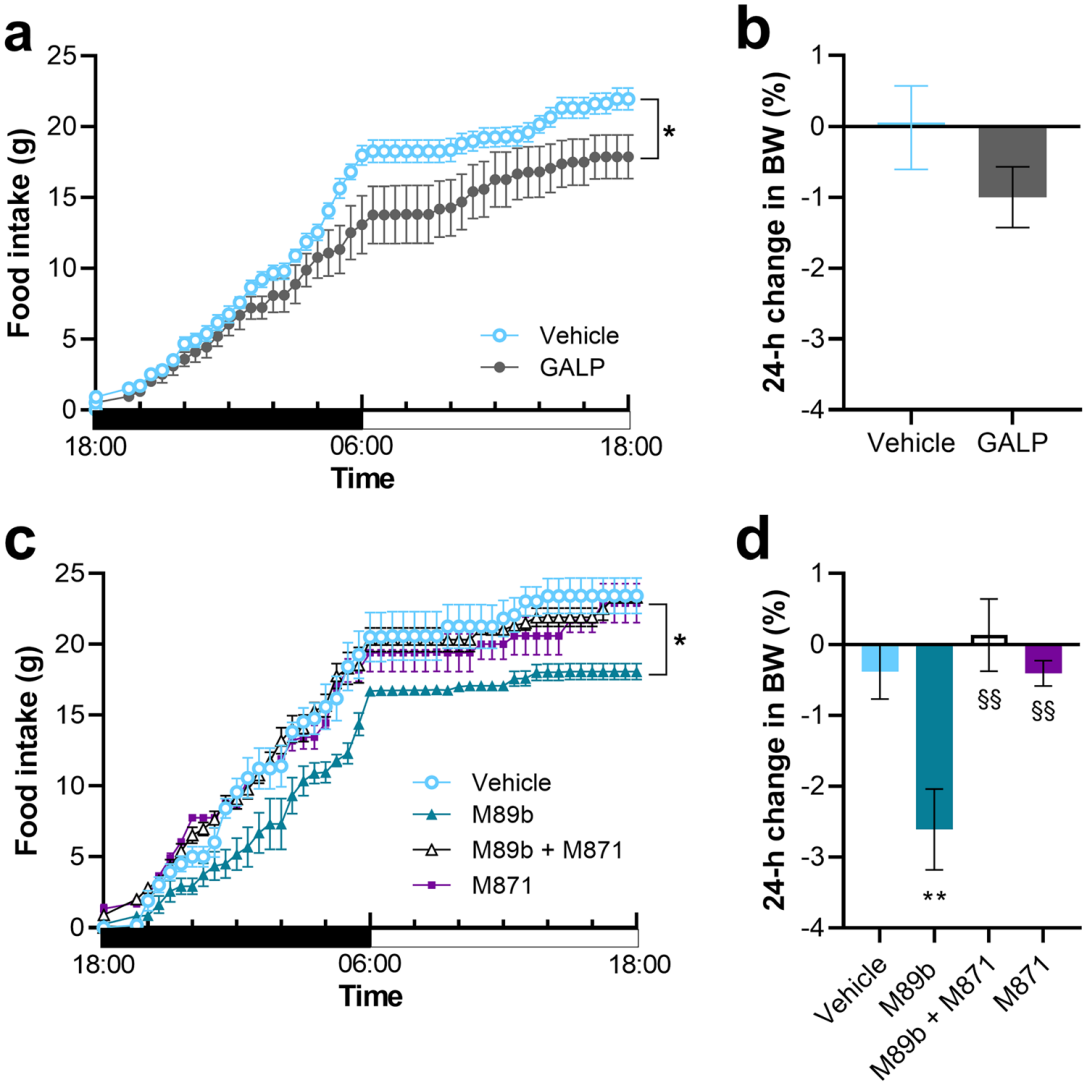
Intranasally administered M89b depressed food intake and resulted in a reduced body weight (BW) in rats. A 24-h food intake was measured in non-fasted rats. During the nighttime period (black horizontal bar), intranasal GALP (17.5 nmol) slowly exerted a significant anorexigenic effect (**a**) without changing the body weight (**b**). Intranasal M89b (25 nmol) significantly reduced the 24-h food intake in rats. Co-administration of M871 (219 nmol) completely abolished the anorexigenic effect of M89b. M871 alone had no effect on food intake (**c**). Compared to vehicle, M89b resulted in a significant reduction of the body weight at 24-h post-treatment, whereas intranasal application of M89b + M871 or M871 alone did not change the body weight (**d**). Data are represented as means ± SEM (*n* = 6–9). Food intake (**a**, **c**): *p < 0.05, repeated-measures two-way ANOVA with Tukey’s post-test. Body weight change (**b**, **d**): body weight presented as % change at 24 h compared to the initial body weight. **p < 0.01 vs. vehicle, §§*p* < 0.01 vs. M89b, one-way ANOVA with Tukey’s post-test

In accordance with the literature [52], intranasal administration of GALP (17.5 nmol) as a positive control significantly reduced 24-h food intake in non-fasted young adult rats by 19%. Repeated-measures ANOVA showed a significant effect of treatment on cumulative food intake during the course of 24 h (*F*(1,13) = 5.305; *p* = 0.038) (Fig. 6a). The test also indicated an interaction between time and treatment (*p* < 0.001). The 24-h body weight showed a trend of reduction, but this change did not reach statistical significance upon GALP treatment (*p* = 0.0247) (Fig. 6b). A more pronounced anorexigenic effect (an overall 23% reduction of 24 h food intake compared to vehicle) developed upon intranasal administration of M89b (25 nmol) (Fig. 6c). The reduction of food intake appeared after some delay. To elucidate whether the effect of M89b on cumulative food intake is indeed mediated via GAL_2_R, we co-administered the GAL_2_R antagonist M871 [54]. Administration of M871 (219 nmol) alone did not influence food intake (Fig. 6c). Combined administration of M89b and M871 effectively prevented the anorexigenic effect of M89b. Repeated-measures ANOVA revealed a significant effect of treatment on cumulative food intake during the course of 24 h [*F*(3,20) = 6.410; *p* = 0.003]. The test also indicated an interaction between time and treatment (*p* < 0.001). Tukey’s post hoc test showed significant differences between M89b- vs. all other groups (vehicle-, M871-, and M89b + M871-treated groups: *p* = 0.006, *p* = 0.021, and *p* = 0.008, respectively). Remarkably, intranasal M89b also resulted in a significant reduction of body weight (Fig. 6d). At 24 h post-treatment, M89b-treated animals have lost 2.6% of their initial body weight (*p* = 0.009 vs. vehicle), while other treatment groups exhibited no body weight change.

## Discussion

The functions of galanin-related peptides, such as galanin, GALP, and spexin, are mediated by GALRs. Over the last two decades, a wide range of GALR agonists and antagonists have been developed. Most of them displayed limited and/or unknown specificity for the three receptors [3, 55, 56]. For therapeutic application of galanin system-related ligands, receptor specificity is of upmost importance to achieve specific treatment results and to avoid unwanted side effects. Here we succeeded in designing and producing a pyroglutamate- and methyllanthionine-stabilized galanin-related agonist with strict GAL_2_R specificity and highly increased stability. M89b has no activity on GAL_1_R or GAL_3_R, not even at concentrations as high as 10 µM, and its affinity via GAL_2_R is slightly lower compared to wild-type galanin or GAL-(1–15) in most assays.

Since the N-terminal region of galanin is essential for activity, our developmental approach started with a C-terminally truncated galanin analog. Accordingly, the introduction of a methyllanthionine before position 13 strongly affected activity on GALRs. Importantly, the first N-terminal amino acid of wild-type galanin seems to have a role in receptor specificity. Its absence appears to abolish the ability of galanin to stimulate GAL_1_R. This observation is in accordance with the fact that GAL-(2–11) is a non-GAL_1_R selective GALR agonist [57, 58]. The introduction of a pyroglutamate at position 1 of our galanin analog strongly increased GAL_2_R affinity. Furthermore, in silico analyses indicated that the binding cavity of GAL_2_R is about twice the size of that of GAL_1_R and about three times the size of that of GAL_3_R [59]. The relative bulkiness of the introduced pyroglutamate and methyllanthionine might therefore contribute to the GAL_2_R specificity of M89b.

As expected, introduction of the pyroglutamate and the methyllanthionine clearly protected against peptidase-mediated breakdown and resulted in greatly increased stability of M89b. In an in vitro stability test using 5% serum, which contains many different peptidases [60, 61], M89b showed a half-life of over 24 h, which is up to three times longer compared to a recently discovered spexin-based GAL_2_R-specific agonist [21]. The increased stability in serum of the latter agonist was suggested to be due to the presence of D-amino acids. In contrast, M89b contains a D,L-methyllanthionine. Importantly, we could also demonstrate increased in vitro stability of M89b in rat CSF and brain plasma, which supports the in vivo activity of the peptide after administration to the brain.

Additionally, Reyes-Alcaraz et al. observed that Thr3 of wild-type galanin is critically important for activation of GAL_2_R and GAL_3_R [21]. However, in our present study, we demonstrated that a Thr3 to Asn3 mutation does not substantially influence GALR activity. Furthermore, our findings show that, after peripheral administration, M89b was not found in brain tissue or urine, the latter indicating that M89b is not immediately excreted via the kidneys. The peptide is not very cationic, which may explain the absence of detectable excretion. Alternatively, breakdown occurs prior to excretion and/or the peptide is degraded by the liver.

Besides the spexin-based GAL_2_R agonist, the M1160 peptide is the only other ligand developed so far as having strict GAL_2_R specificity, as all others still retain activity on GAL_1_R and/or GAL_3_R at high concentrations [62, 63]. Compared to wild-type galanin, M1160 displays a relatively low affinity toward GAL_2_R. Importantly, no data on the stability of M1160 are available [62]. Nevertheless, M1160 showed antidepressant effects after intracerebroventricular (icv.) application in mice [62]. Spexin-based GAL_2_R-specific variants exerted anxiolytic effects in mice [21] and therapeutic activity for mood and feeding disorders [56] after icv. administration. Interestingly, peripherally acting non-peptidergic agonists with preferential binding to GAL_2_R over GAL_1_R have been discovered and showed analgesic properties in rodent models of inflammation, neuropathic pain, acute pain, and epilepsy [55, 64, 65]. The selectivity of these agonists with respect to GAL_3_R has not been reported.

Taken together, the strict GAL_2_R specificity and the high in vitro stability together with the enhanced in vivo stability strongly support the clinical usefulness of M89b. Indeed, in the present study, we provide evidence that M89b is functionally active in vivo.

The galanin system plays a role in feeding, as GALRs are expressed in brain regions important for appetite control [2]. Galanin was shown to consistently stimulate food intake following acute central injection into the hypothalamic paraventricular, lateral, and ventromedial nuclei, as well as the central nucleus of the amygdala, producing a rapid increase in the feeding response. Furthermore, the literature suggests that galanin activates acute feeding behavior rather than suppressing satiety [9–12]. Although neither GAL_1_R- nor GAL_2_R-KO mice displayed any marked phenotype related to feeding behavior [66, 67], an icv.-administered selective GAL_1_R agonist, M617, induced consumption of high-fat milk in rats, whereas a selective GAL_2_R agonist, M1153, did not [68]. However, it has to be noted that both of these two agonists retain some activity on other GALR subtypes. Interestingly, GALP was shown to reduce food intake in wild-type mice, as well as in GAL_1_R- and GAL_2_R-KO mice [69], indicating that the effect of GALP on feeding behavior might not be exclusively mediated by GALR signaling. Furthermore, an anti-obesity effect of GALP after intranasal administration was shown [52]. Thus, we used GALP in the present study as a positive control and confirmed its anorexigenic effect resulting in a trend of weight loss that did not reach statistical significance. Importantly, we found that intranasally delivered M89b reduced acute food intake and 24-h body weight in rats, thus demonstrating M89b activity in vivo. A possible increase in metabolic rate could contribute to the stronger weight loss in case of M89b administration. The effects on food intake and body weight were abolished by co-administration of the GAL_2_R-specific antagonist M871. To our knowledge, this is the first study showing in vivo activity of a lanthionine peptide upon intranasal application. Furthermore, our data support recent findings on a GAL_2_R-selective spexin analog [56], which indicates that the regulation of food intake by GALP and spexin is mediated by GAL_2_R signaling. However, there are no studies available which tried to block the effects of GALP on food intake with GALR-specific antagonists to prove the involvement of GALRs in GALP-mediated effects. As chronic administration might lead to resistance to treatment, it will have to be elucidated whether chronic administration of M89b reduces the body weight of obese animals. A first hint that this could be the case comes from the observation that intranasal administration of the spexin-based GAL_2_R agonist SG2A for 14 days decreased food intake and body weight [56]. Taken together, these data indicate that, depending on the route of administration and type of receptor activated, GALR signaling might have either orexigenic or anorexigenic effects.

Intranasal delivery of peptides to the brain has been reported for a limited number of peptides [35], including GALP [52]. This method is clearly less invasive than many other routes of peptide administration to the brain. The key requirement of reaching effective levels at the intended brain areas appears to have been met by intranasal delivery of M89b, which reduced food intake and body weight. Further studies using labeled M89b may assess the exact distribution of the nasally delivered peptide.

In conclusion, intranasal delivery of a GAL_2_R-selective agonist offers therapeutic opportunities for treating a variety of brain diseases. GAL_2_R has, for instance, been reported to be neuroprotective in experimental autoimmune encephalomyelitis and a cuprizone model mimicking aspects of multiple sclerosis [70, 71]. A non-functional GAL_2_R in a multiple sclerosis patient has been reported [72], indicating a potential therapeutic role for stimulation of GAL_2_R in this disease. The current study raises the possibility of an anti-obesity effect of M89b that could be confirmed via chronic or repeated administration in future studies.

## Supplementary Information

Below is the link to the electronic supplementary material.

Supplementary file1 (PDF 616 KB)

Supplementary file2 (PDF 3506 KB)

Supplementary file3 (PDF 1358 KB)

Supplementary file4 (PDF 462 KB)

Supplementary file5 (PDF 468 KB)

Supplementary file6 (PDF 628 KB)

Supplementary file7 (PDF 507 KB)

Supplementary file8 (PDF 230 KB)

## Data Availability

The raw data supporting the conclusions of this article will be made available by the authors, without undue reservation, to any qualified researcher.
