# Supplementary material for: Intranasal Delivery of a Methyllanthionine-Stabilized Galanin Receptor-2-Selective Agonist Reduces Acute Food Intake
Source: Neurotherapeutics. 2021 Dec 2;18(4):2737–52. doi: 10.1007/s13311-021-01155-x (PMC8804135; doi:10.1007/s13311-021-01155-x)
Supplement: Supplementary file 8 — Supplementary file8 (PDF 230 KB) [file 13311_2021_1155_MOESM8_ESM.pdf]

SUPPLEMENTARY MATERIAL

to

Intranasal delivery of a methyllanthionine-stabilized galanin receptor-2-selective agonist reduces acute food intake

Anneke Kuipers[1], Márta Balasko[2], Erika Pétervári[2], Andreas Koller[3,4], Susanne M. Brunner[3,4], Gert N. Moll[1,5], Barbara Kofler[3]*

Supplementary methods

In vitro *stability in brain plasma*

Sprague dawley rat brain lyophilized powder was purchased from MyBioSource (San Diego, USA). One gram brain powder was reconstituted in 10 ml PBS, 0.5 ml 2 M Tris (pH=8), 100 µl Triton X-100 and 10 mg SDS. The solution was centrifuged and the supernatant was collected, aliquoted and stored at -20ºC until usage. To assess the stability, 10 µM GAL-(1-15) and 10 µM M89b were incubated in 1% brain plasma buffered with 200 mM phosphate buffer (pH 7.4) at 37°C for up to 6 hours. At various time points (0, 15, 30, 60, 120 and 360 min), 200 µl sample was quenched with 10 µl 50% TFA and 20 µl 100% ACN. Samples were centrifuged and kept on ice. Full-length peptides were quantified by using a C12 column (C12 RP 250 x 4.60 mm column, Phenomenex) on a JASCO HPLC system and applying a gradient of 20-90% ACN in 0.1% TFA for 30 minutes. Peptides were detected at 280 nm. A280 areas in chromatograms of the corresponding GAL-(1-15) and M89b full-length peptide peaks were used to determine the amount of peptide left after incubation with cerebrospinal fluid at the different time points. Peptide peaks were determined using JASCO-Borwin Chromatography software (v1.50).

Supplementary figures

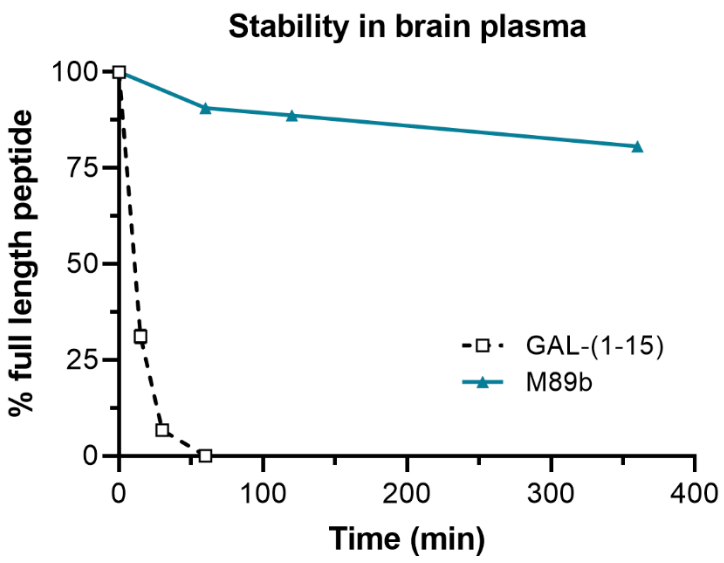

Supplementary Figure S1 M89b has increased *in vitro* stability in brain plasma. Degradation of M89b in comparison to GAL-(1-15) in 1% rat brain plasma over time. Data represent means ± SEM. n=3.
